# Community-Based Participatory Research Integrates Behavioral and Biological Research to Achieve Health Equity for Native Hawaiians

**DOI:** 10.3390/ijerph13010004

**Published:** 2015-12-22

**Authors:** Claire K. M. Townsend, Adrienne Dillard, Kelsea K. Hosoda, Gregory G. Maskarinec, Alika K. Maunakea, Sheryl R. Yoshimura, Claire Hughes, Donna-Marie Palakiko, Bridget Puni Kehauoha, Joseph Keawe‘aimoku Kaholokula

**Affiliations:** 1 Department of Native Hawaiian Health, John A. Burns School of Medicine, University of Hawai’i at Mānoa, 651 Ilalo Street, MEB 307L, Honolulu, HI 96813, USA; clairemt@hawaii.edu (C.K.M.T.); khosoda@hawaii.edu (K.K.H.); gregorym@hawaii.edu (G.G.M.); amaunake@hawaii.edu (A.K.M.); kaholoku@hawaii.edu (J.K.K.); 2 Kula no na Po‘e Hawai‘i, P.O. Box 2368, Honolulu, HI 96823, USA; kula.papakolea@gmail.com (A.D.); punikekauoha@gmail.com (B.P.K.); 3 Kōkua Kalihi Valley Comprehensive Family Services, 2239 N. School Street, Honolulu, HI 96819, USA; sryoshimura@gmail.com; 4 Hawai‘i Maoli, Association of Hawaiian Civic Clubs, P.O. Box 3866 Honolulu, HI 96812, USA; hughes1926@aol.com; 5 Ke Ola Mamo, Dillingham Plaza, 1505 Dillingham Blvd #205, Honolulu, HI 96817, USA; dpalakiko@keolamamo.org

**Keywords:** community-based participatory research, epigenomics, diabetes self-management, Native Hawaiians

## Abstract

Native Hawaiians bear a disproportionate burden of type-2 diabetes and related complications compared to all other groups in Hawai‘i (e.g., Whites, Japanese, Korean). Distrust in these communities is a significant barrier to participation in epigenetic research studies seeking to better understand disease processes. The purpose of this paper is to describe the community-based participatory research (CBPR) approach and research process we employed to integrate behavior and biological sciences with community health priorities. A CBPR approach was used to test a 3-month evidence-based, diabetes self-management intervention (N = 65). To investigate the molecular mechanisms linking inflammation with glucose homeostasis, a subset of participants (n = 16) provided peripheral blood mononuclear cells. Community and academic researchers collaborated on research design, assessment protocols, and participant recruitment, prioritizing participants’ convenience and education and strictly limiting the use of the data collected. Preliminary results indicate significant changes in DNA methylation at gene regions associated with inflammation and diabetes signaling pathways and significant improvements in hemoglobin A1c, self-care activities, and diabetes distress and understanding. This study integrates community, behavioral, and epigenomic expertise to better understand the outcomes of a diabetes self-management intervention. Key lessons learned suggest the studies requiring biospecimen collection in indigenous populations require community trust of the researchers, mutual benefits for the community and researchers, and for the researchers to prioritize the community’s needs. CBPR may be an important tool in providing communities the voice and protections to participate in studies requiring biospecimens.

## 1. Introduction

In Hawai‘i, Native Hawaiians (NH) have an overweight/obesity prevalence of 73% compared to Whites (56%) [1]. Correspondingly, NH have a greater burden of obesity-related diseases, such as type 2 diabetes, heart attacks, and coronary heart disease, compared to Whites [1]. Native Hawaiians (11.1%) have a prevalence of diagnosed diabetes over twice that of Whites (5.1%) and are diagnosed with diabetes at younger ages, 45.9 years in NH compared to 50.4 years in Whites [1]. To address these obesity-related disparities in Hawai‘i, a community-based participatory research (CBPR) partnership called the PILI ‘Ohana Project (POP) was formed in 2005. Community-based participatory research is an approach to research which seeks to equally involve community and academic partners throughout the research process in a co-equal, co-learning partnership [2]. Community and participant engagement in research, especially research with populations that have a history of mistreatment, is increasingly viewed as important for conducting ethical research [3]. This approach to research is increasingly being used with indigenous, as well as other ethnic populations. Through the formation of co-equal partnerships, historically disenfranchised populations are now able to exert control over the research conducted in their communities [4]. Additionally, the research being conducted in more relevant to the communities’ health needs.

The POP CBPR partnership is comprised of academic investigators from the Department of Native Hawaiian Health at the John A. Burns School of Medicine (JABSOM) and community investigators from four community-based organizations: Hawai‘i Maoli the nonprofit arm of the Association of Hawaiian Civic Clubs that promotes the social and economic self-sufficiency of NH communities, Kula no na Po‘e Hawai‘i (KNNPH) a non-profit organization that provides health and educational activities for members of the three Hawaiian Homestead communities, Ke Ola Mamo the NH Health Care System for the island of O‘ahu providing health services to primarily low income NH, and Kōkua Kalihi Valley Comprehensive Family Services a community-owned non-profit organization providing health services to Pacific Islanders and immigrant Asians. The POP has successfully developed and tested healthy culturally tailored, evidence-based health lifestyle interventions for Native Hawaiians and other Pacific Islanders living in Hawai‘i. For more details regarding this decade-long, CBPR partnership, see articles by Nacapoy, *et al.* [5] and Kaholokula, *et al.* [6].

In addition to behavioral lifestyle programs, described by Kaholokula, *et al.* [7] and Mau, *et al.* [8], the POP tested and adapted a diabetes self-management program for NH, as well as other Pacific Islanders in Hawai‘i. The intervention, entitled Partners in Care (PIC), involves 12 weekly, 1-h group meetings. Sinclair, *et al.* [9] provide more details about the PIC lessons and foci. Briefly, the curriculum provides an overview of diabetes and self-management activities, American Diabetes Association recommended standards of care, strategies to improve patient-provider communication, information about diabetes medications, and ways to avoid diabetes-related complications. This intervention was designed for delivery in community settings by community peer educators. In a previous, randomized delayed-intervention-control design testing the adapted PIC, a significant reduction in hemoglobin A1c was found based on both an intent-to-treat analysis (−1.1% ± 0.2) and a complete case analysis (−1.6% ± 0.2) in a cohort of NH, which included other Pacific Islanders as well [9].

Culturally-tailored, evidence-based, behavioral interventions are important in addressing obesity-related health disparities effecting NH. Additionally, research into the biological processes affected by a behavioral intervention can provide a more complete understanding of the intervention’s effects and uncover new strategies to eliminate health disparities. Examination of the molecular mechanisms linking chronic inflammation with glucose homeostasis in people with diabetes is one such area of research. Growing evidence suggest that the accumulation of inflammatory cells in the vessel wall is a key feature of diabetes-related medical complications, such as heart disease. However, little is known about the molecular mechanisms that underlie this chronic inflammation phenotype. Recent studies demonstrate nutritional factors modify the inflammatory characteristics of specific immune cells, such as monocytes, in patients with diabetes, indicating that chronic inflammation may have a non-genetic basis [10]. Indeed, preliminary epigenome-wide association data identified selected DNA methylation variable positions that precede diabetes diagnosis [11]. Together, these studies suggest a link between diet-induced changes in epigenetic processes that modify inflammatory characteristics of immune cells and the diabetic condition. It has been suggested that the application of such epigenome-wide association data to healthcare could revolutionize current studies of human diseases and advance diagnostic, preventive, and treatment strategies [12]. To examine this “bio-behavioral” link, the collection of biospecimens is necessary.

As with most indigenous populations, NH are distrustful of research. Native Hawaiians’ experience with the harm posed by research, dates back to the 1800s. At this time, individuals diagnosed with Hansen’s disease were exiled to a remote part of the island of Moloka‘i. During their exile, a government physician conducted studies to discover the mode of transmission [13]. This mistreatment has continued. Researchers expect that a large Native Hawaiian family carried a rare genetic condition. While this research was approved, participants did not provide informed consent nor told of the results. Additionally, no consideration was made for genetic testing, counseling, or treatment [14]. Today, NH participants in research reported feeling as though they were “used as guinea pigs” to advance the researcher’s career, their views were misinterpreted, and there were no benefit to their community [15,16,17,18]. This distrust is particularly strong for research involving the collection of biospecimens [15]. Academic research performed in NH communities, without community input, can be viewed as contributing to the legacy of colonization and exploitation [19]. An example of this was the 2003 proposal by the University of Hawai‘i to patent the NH genome, which was strongly opposed by the NH community [20]. Additionally, there is a history of, and resulting wariness toward, “helicopter research” in which academic investigators “fly” into NH communities, conduct their research, and “take off”, leaving nothing of immediate or tangible value to the communities.

It is in the environment of distrust and past abuse that the POP was first developed. The POP, a long-standing CBPR partnership with established trust between the NH community and academic investigators, realized the potential value of epigenetics in advancing the science of health disparities and the importance of coupling epigenetic studies with intervention research to provide immediate, tangible benefit to the community. Thus, the POP community-academic partnership developed a study to examine the impact of PIC at the behavioral, clinical, cellular, and molecular levels wherein we examined the inflammatory characteristics and epigenomic profiles of immune cells from NH with diabetes. Prior to an examination of the study’s results, it important to understand the process through and partnerships in which this study was conducted. Therefore, the purpose of this paper is to describe the CBPR approach and data collection process we employed to ensure active decision-making and leadership by community partners, full disclosure of the study benefits and risks to participants, and input from the participants themselves. Through understanding our CBPR partnership and process we developed for integrating behavioral and biological sciences with community health priorities, we hope that researchers will be able to apply our lessons learned and recommendations to their work with other communities.

## 2. Research Development and Implementation Process

### 2.1. Development

The POP allowed a new investigator from the Department of Native Hawaiian Health to present an overview of his research in the areas of epigenetics and epigenomics at their monthly Intervention Steering Committee meeting. These meetings are used to monitor current activities, review past successes and challenges, and create plans for future endeavors. Members of the Intervention Steering Committee include the academic and community Principal Investigators, the project coordinators, and research assistants affiliated with the POP projects. This new investigator is a NH. Community investigators asked a variety of questions at this meeting and went on to discuss what they learned with other community stakeholders. Over several months, this new investigator attended POP monthly meetings to follow-up on potential research activities. During this time an opportunity arose to test a community investigators’ idea, which was whether a diabetes support group delivered post-PIC intervention would improve or maintain the gains made during the PIC intervention. It was also an opportunity for inclusion of epigenetic research. The POP successfully received a research development award as part of a NIH-supported, infrastructure grant with two complementary specific aims: (1) to test a semi-structured social support component as a supplement to an evidence-based diabetes self-management intervention; and (2) to investigate the impact of this community-based diabetes intervention on the epigenomes of diabetic NH and other Pacific Islander participants at risk for diabetes-related complications.

### 2.2. Implementation

To address the first specific aim, the POP conducted a randomized controlled trial testing the effects of a 3-month, 6-session, semi-structured support group (SSG) against standard follow-up care. Sixty-five NH and other Pacific Islanders received the PIC intervention and were then randomized to either SSG or standard follow-up. The SSG was designed to reinforce the self-care skills taught in PIC and address any new or pending concerns. Professionals with diabetes-specific knowledge (e.g., health psychologist, physician) conducted half of the support group sessions while trained community peer educators conducted the remaining groups.

The second specific aim included epigenomic analyses, which required biospecimens. The POP met several times to determine the most appropriate protocol for educating potential participants about this component, obtaining informed consent, providing incentives, collecting samples, and debriefing once the study was complete. The informed consent process included a full disclosure of the collection, use, and disposal of samples, which was limited to the study’s specific research questions. The investigators also held an informational meeting for community participants. Separate from the intervention study, this epigenetic component was reviewed and approved by two Institutional Review Boards: University of Hawai‘i and Papa Ola Lōkahi Native Hawaiian Health Board.

A statistically-powered sample size of 16 was determined empirically using preliminary epigenomic data collected prior to this study. This determination was only pertinent to the epigenomic aspect of the study. Community facilitators, via word of mouth and flyers at community centers, conducted participant recruitment. All participants enrolled in the larger PIC trial were eligible for the epigenetic aspect of the study and the first 16 participants to express interest in participating in the the epigenetic aspect of the study would be enrolled. Participants were made aware of the epigenomic assessment component during the screening for the PIC intervention. The first 16 participants who enrolled in the PIC study were also interested in participating in the epigenomic assessment component of the study. All participants were from KNNPH, a non-profit organization serving three urban Hawaiian Homesteads. After expressing interest in the epigenomic assessment component of the study, all participants received education on the study, which included basic instruction on epigenetics and how concepts of Native Hawaiian health relate to this area of science. This education preceded consent, so that potential participants could make an informed decision regarding their participation in the study.

All of the initial 16 participants who expressed interest in the epigenomic component of the study and received education about epigenomics and the study, provided informed consent to being part of the study. These participants donated three tablespoons of whole blood pre- and post-intervention, from which we collected peripheral blood mononuclear cells (PBMCs). See Table 1 for summary demographic and baseline clinical data on these participants. Certified phlebotomists collected the blood samples at KNNPH, transported the samples to their lab, and isolated PBMCs within two hours of sample collection to preserve cell viability. Academic investigators were on-site throughout sample collection to address any concerns or questions from participants. The time burden on participants for this blood donation process was approximately 30 min. From the cryopreserved PBMCs, we enriched for a specific subpopulation of immune cells to perform epigenomic analyses and evaluated the inflammatory characteristics of these cells. After analyzing the data, academic and community partners discussed the results and decided that participants should have an opportunity to learn the results and provide feedback prior to further dissemination.

**Table 1:** Participants’ baseline socio-demographic, behavioral, and biological characteristics.

Variable	*n* = 16
Age (years)	49.56 ± 10.15
Sex	
Female	6 (37.50)
Ethnicity	
Hawaiian	16 (100)
Education	
High school	13 (81.25)
>High school	3 (18.75)
Marital status	
Never	2 (12.50)
Currently	13 (81.25)
Disrupted	1 (6.25)
Weight (kg)	104.82 ± 28.85
BMI (kg/m^2^)	36.42 ± 6.64
HbA1c (%)	8.97 ± 1.62
Cholesterol (mg/dL)	183.25 ± 43.03
LDL Cholesterol (mg/dL)	87.67 ± 31.20
HDL Cholesterol (mg/dL)	34.82 ± 6.31
Triglycerides (mg/dL)	349.81 ± 210.76
Systolic blood pressure (mmHg)	125.73 ± 12.54
Diastolic blood pressure (mmHg)	73.77 ± 8.77
Diabetes care profile score	3.00 ± 0.71
Problem areas in diabetes score	44.45 ± 18.47
Summary of diabetes self-care activities score	16.25 ± 5.04

Notes: Body Mass Index is abbreviated as BMI, High density lipoprotein as HDL, and Low-density lipoprotein as LDL. A1c is the measure of glycated hemoglobin. Data shown as Mean ± SD or n (%).

### 2.3. Follow-Up

Community investigators invited all of the participants enrolled in the epigenomic assessment component of the study to a presentation by the POP to summarize the clinical, inflammatory, and epigenomic data. Fifty percent of the participants chose to attend. Education and sharing of results were conducted via PowerPoint presentations and open forum discussions with study participants in a community setting. The PI of the study conducted this session with the assistance of the PIC community peer educator. At the recommendation of community leaders, as well as participants of the study, a newsletter presenting the general results of the study and highlighting the research collaboration with the community was created and delivered to the Hawaiian Homestead communities from which the participants were recruited.

Following a presentation of the results of the study by our academic investigator, we conducted a one-hour focus group with these eight PIC participants. An investigator with expertise in qualitative data collection and analysis facilitated the focus group. This investigator was not part of the study proper to better enable an unbiased discussion. To frame the conversation, the investigator used a semi-structured discussion guide and to encourage participants to fully express their views and ensure understanding, he also used spontaneous probes. All eight focus group participants, five males and three females, expressed satisfaction with the epigenomic experiment, the PIC intervention, and the summarized results presented. One participant praised KNNPH for bringing this research opportunity to their community. None of the participants expressed concern with regard to the collection of biological specimens. In fact, the only barrier to participation that participants cited was the time involved in the blood draw and health assessment. Interestingly, some participants stated that giving blood, even in this context, was considered a gift and that they chose to participate in this study knowing that the samples collected were going to benefit others. Their willingness to participate is indicative of their trust in KNNPH and in the academic investigators. The results of the study provided evidence that the behavioral changes they made during the PIC intervention (e.g., increased frequency of diabetes self-care activities such as checking blood glucose and feet and increased physical activity) were positively impacting their health. Participants believed that these results reinforced the behavioral changes they had made to improve the management of their diabetes. One participant stated, “It [the results] shows that you’re doing what you need to be doing, so if you keep it up, you’ll get better.” Participants perceived these results as very motivating and encouraging with regards to their continued use of skills learned from the PIC intervention to improve their diabetes self-management. One participant stated, “If this isn’t motivating for them, I don’t what else can motivate them.” Therefore, participants supported recommendations that these results be widely disseminated.

A comprehensive review of the data analysis and results is outside the scope of this paper. Briefly, consistent with a previous report [9], results of this study suggested that molecular changes, including modifications to inflammation likely mediated by epigenetic processes, preceded the clinical improvements of glycemic control as measured by hemoglobin A1c levels in PIC participants. The overall perception of the study was positive amongst both participants and researchers. The trust established between these specific Hawaiian Homestead communities and academic investigators laid the foundation for future collaborations on biological studies with NH communities that require biospecimens, especially when it is linked to immediate and tangible benefits for community members.

## 3. Lessons Learned

This study used a CBPR approach to investigate the impact of a culturally-tailored, evidence-based diabetes self-management intervention on the epigenetic regulation of inflammatory phenotypes of immune cells from NH participants. Community and academic investigators collaborated on the research design, intervention, and participant education and assessment protocols. Through numerous meetings and discussions, a protocol for participant assessment that respected the autonomy, dignity, and cultural beliefs and values was developed. This protocol also prioritized logistical considerations and education of participants, strict limits on the use of the samples collected, and the community’s ownership of the data and control over dissemination.

### 3.1. Trust

Kula no na Po‘e Hawai‘i has a responsibility to protect the interests, privacy, and autonomy of the community members they serve. This responsibility is an important consideration in KNNPH’s decision to engage in any research and why they were skeptical of moving too quickly into research involving biospecimen collection. Prior to their involvement in the POP, KNNPH declined to participate in research that involved blood collection due to a lack of relationship and trust with those proposing the research. The nurturing of a CBPR partnership by the POP over the last decade allowed for trust to develop between Department of Native Hawaiian Health and KNNPH. Through participating in the POP, KNNPH has brought healthy lifestyle interventions and resources into their community, garnering community trust on research issues. It was in this context that the study described here was proposed. The academic and community investigators had open, honest conversations about community organization limitations (e.g., resources, recruitment potential) and the benefits of the research (e.g., funding, potential new way to examine diabetes). Additionally, the fact that the investigator proposing the biological research was a NH with community connections and who presented the information in a manner accessible to the community helped to build on the foundation of trust.

### 3.2. Mutual Benefit

Research undertaken should benefit the community and science alike. The POP has a decade-long history of addressing the health needs of NH communities in Hawai‘i. The intervention research conducted by POP provided tangible and sustainable benefits for community members (e.g., weight loss, improved diabetes management, skills in designing and implementing health promotion strategies) because they are community-placed. Alone, a study examining the biological basis of inflammation and glucose homeostasis in diabetic individuals would have had little immediate or noticeable benefits to the community. However, linking such a study with the delivery of a proven diabetes self-management intervention and testing a community recommended social support component allowed for mutually beneficial outcomes—advancing our scientific understanding of effective interventions and their biological and behavioral outcomes, while building the community’s capacity to address their own health concerns on their own terms.

### 3.3. Prioritize Community

We also created a protocol for collecting biospecimens that was acceptable to the community investigators and participants. Meetings to discuss study protocol, participant assessment, blood draws, program delivery and presentation of the findings all occurred within the community setting. A variety of issues were considered when deciding on the biospecimen collection procedures, including the two-hour time restraint from blood collection to processing of viable cells and phlebotomist needs. However, priority was given to participants resulting in a protocol that was the most convenient, comfortable, and culturally and physically safe for participants. For example, this meant adjusting the usual daytime schedules of blood sample processing for investigators isolating PBMCs, which meant extra hours of work late at night.

## 4. Recommendations

The integration of clinical, behavioral, and biological sciences is important in effectively addressing obesity-related disparities in health disparate populations. However, past abuse and discrimination, which led to distrust and disengagement, have stymied such integration in health disparities research with NH communities. Community-based participatory research may be an effective approach to establish trust and integrate clinical, behavioral, biological, and community expertise. We recommend special attention be paid to several aspects of the CBPR approach when proposing and conducting studies involving biospecimen collection and analyses. These recommendations are consistent with those presented by Vawer, *et al.* [21] and Hiratsuka, *et al.* [22].

### 4.1. Communicate Openly and Honestly

The academic and community investigators need to have open and honest communication. Communication should not be limited to one or two formal meetings but should be ongoing throughout the study proposal and research process and should extend to community members. Having open communication allows for concerns and potential misunderstandings to be expressed and addressed quickly throughout the study. The co-learning and co-equality promoted by CBPR facilitates this open, honest communication between partners.

### 4.2. Devote the Time

Community-based participatory research can be more time consuming than traditional approaches to research, which in our experience is largely due to logistical issues. Both community and academic investigators should be prepared to spend time building their relationship. Academic investigators should expect to spend time explaining the proposed research and addressing concerns from community members, and approach these interactions as opportunities to continue to build the research relationship. Time must also be spent on soliciting and incorporating feedback from participants and community leaders prior to the publication or presentation of the results. In fact, the community partners need to be co-authors and co-presenters on all dissemination activities. Overall, academic investigators need to move at whatever pace is comfortable for the community participating in the study and not to rush toward reaching research objectives in isolation.

### 4.3. Support Indigenous and Minority Researchers

Supporting and mentoring emerging investigators from indigenous groups and other ethnic minorities can improve the science and the relationships with indigenous and minority communities, broaden the types of research conducted, and build on community wisdom and cultural perspectives and knowledge. Although relationships and trust can be built by academic investigators from different ethnic backgrounds, this process can proceed more rapidly and be nurtured organically when the academic investigators have ties to the community with which they conduct the research. Additionally, researchers who are from the communities that they study may be better able to identify research-related risks for their communities [23].

### 4.4. Balance Community and Academic Benefits

Finally, there is a need to ensure mutual and balanced benefits of a research activity. The benefits to academic investigators are numerous (e.g., publications, funding opportunities, and promotion and tenure). The benefits to community can also be numerous when done right, such as mobilizing and equipping a community to effectively address a community concern. Through fluid communication and shared values, collaboratively conceiving the research, recognizing the shared missions of each party, and respecting the perspectives from which each party bases their decisions enhances the recognition of community needs alongside that of academic partners. This will support the development of an organic relationship and mutual trust, allowing for advances in biomedical and behavioral research as well as in community development.

## 5. Conclusions

The integration and synthesis of clinical, behavioral, and epigenomic expertise to comprehensively examine behavioral influences of health outcomes of culturally-relevant intervention programs is unprecedented in Hawai‘i. The CBPR approached used in this study enabled academic researchers to articulate to Hawaiian Homestead community members and stakeholders that the etiology of diabetes includes an epigenetic basis and enabled community stakeholders to continue to address diabetes disparities in their communities. Based on the positive experiences of the community and academic investigators, as well as community stakeholders and participants, we believe that the process used in this study was successful. However, it should be noted that this study lacked a comparison group. Therefore, we do not know what the experience for community and academics would have been in without the significant community engagement. As noted above, there have been previous attempts by other investigators to conduct research involving biospecimen collection in the POP communities that were denied. These investigators, while well intentioned and back by rigorous science, lacked relationships with the community, did not engage the community until the research agenda has set, and were unsuccessful in garnering community support for their project. We believe that the mutual benefits achieved by this study have strengthened the engagement between academic researchers and community stakeholders. Continued adherence to the lessons learned and recommendations describe here will be characteristic of our future CBPR endeavors. It is the intention of the authors that this strengthened relationship will translate into future CBPR to address NH health disparities through a combination of biological and behavioral sciences and community health priorities.
